# MicroRNA-155—at the Critical Interface of Innate and Adaptive Immunity in Arthritis

**DOI:** 10.3389/fimmu.2017.01932

**Published:** 2018-01-05

**Authors:** Stefano Alivernini, Elisa Gremese, Charles McSharry, Barbara Tolusso, Gianfranco Ferraccioli, Iain B. McInnes, Mariola Kurowska-Stolarska

**Affiliations:** ^1^Institute of Rheumatology – Fondazione Policlinico Universitario A. Gemelli, Catholic University of the Sacred Heart, Rome, Italy; ^2^Institute of Infection, Immunity and Inflammation, University of Glasgow, Glasgow, United Kingdom; ^3^Rheumatoid Arthritis Pathogenesis Centre of Excellence (RACE), Glasgow, United Kingdom

**Keywords:** microRNA-155, inflammation, antibody production, arthritis rheumatoid, autoimmunity

## Abstract

MicroRNAs (miRNAs) are small non-coding RNAs that fine-tune the cell response to a changing environment by modulating the cell transcriptome. miR-155 is a multifunctional miRNA enriched in cells of the immune system and is indispensable for the immune response. However, when deregulated, miR-155 contributes to the development of chronic inflammation, autoimmunity, cancer, and fibrosis. Herein, we review the evidence for the pathogenic role of miR-155 in driving aberrant activation of the immune system in rheumatoid arthritis, and its potential as a disease biomarker and therapeutic target.

## Introduction

Epigenetic modification represents an important mechanism of regulating gene expression that enables cells to respond appropriately to a changing environment. The mechanisms of epigenetic modification include, e.g., acetylation of histones, methylation of gene promoters, and silencing of mRNA transcripts by microRNAs (miRNA) (1). miRNAs are a family of short, non-coding RNAs, acting at the posttranscriptional level (2, 3), that fine-tune mRNA transcription (4). More than 2,000 miRNAs have been identified in humans, and computational predictions show that these regulate the expression of approximately 60% of all human protein-coding genes (5). miRNAs are pleotropic; a single miRNA can regulate a cellular response by targeting multiple components of a biological pathway (2). More than 100 different miRNAs are expressed by cells of the immune system together influencing the pathways that control the development and function of cells of innate and adaptive immune responses (2, 6, 7). Identifying disease-specific miRNAs improved the understanding of molecular pathways involved in diseases, and provided an evidence-base for new therapeutic strategies, e.g., in cancer (8) and tendinopathy (9).

Rheumatoid arthritis (RA) is an autoimmune disease that affects approximately 1% of the global population and leads to progressive loss of joint function (10). Currently, more than half of RA patients do not achieve sustained drug-induced disease remission (11), which constitutes an important clinical unmet need. A better understanding of the disease process is required to improve the treatment options for patients resistant to current therapeutics and to provide an evidence base for personalized medicine.

A decade of research has shown that aberrant expression of miRNAs underlies the immune response and stromal cell activation in RA (2, 12). miR-155 is a master-regulator of the immune response and the aim of the present review is to discuss its role in the immuno-pathogenesis of RA and its potential as a disease activity biomarker and therapeutic target.

## miRNA Biogenesis and Mechanism of Action

MicroRNAs are ~23 nt small non-coding RNAs that regulate mRNA expression at the posttranscriptional level by directing mRNA degradation or translational repression. They fine-tune expression of their target genes (by approximately 1.2-fold to fourfold) and correspondingly affect biological pathway function (2). miRNAs bind complementary seed-region sequences in the 3′ untranslated regions (UTRs) of specific target mRNAs leading to the repression of protein production. Each miRNA has the potential to repress many target mRNAs, often in the same molecular pathway, highlighting the sophistication of this epigenetic regulation.

MicroRNAs emerge from long primary transcripts (pri-miRNAs) transcribed from independent miRNA coding genes or from introns of protein-coding mRNAs (13, 14). After transcription, pri-miRNAs are capped, poly-adenylated, and then cleaved into ~70 nt hairpin structures (pre-miRNAs) by a nuclear microprocessor complex composed of RNase III-type endonuclease Drosha and the DiGeorge critical region 8 protein. DGCR8 is essential for Drosha activity and is capable of binding single-stranded fragments of the pri-miRNA that are required for proper processing. Pre-miRNAs are then exported from the nucleus to the cytoplasm through Exportin-5 in a GTP-dependent mechanism. In the cytoplasm, pre-miRNAs are then cleaved by Dicer; a RNase III-type enzyme that acts with its cofactor TAR RNA-binding protein 2 resulting in RNA duplexes of ~22–23 nt in length. Subsequently, an argonaute (Ago) protein and a 182-kDa glycine–tryptophan repeat-containing protein bind to these RNA duplexes forming the core of a multi-subunit complex called the miRNA-mediated silencing complex (miRISC). Once assembled into the miRISC, through base-pairing interactions between nucleotides 2 and 8 of the miRNA (the seed region) and complementary nucleotides predominantly in the 3′-UTR of mRNAs, they act as repressor of mRNA translation (15–18).

The miR-155 gene was initially described as the B-cell integration cluster gene (*bic*) in chickens, which induced leucocytosis when activated by a viral promoter insertion that increased *bic* transcription (19). Following this finding, a homologous gene to *bic* was identified in humans and mice (20). Human and murine *bic* cDNA have >70% identity (20). *Bic* is strongly expressed in the thymus and spleen, and can be detected in other tissues, including liver, lung, and kidney (20–22). At the cellular level, analysis of small RNA clone libraries by Landgraf and colleagues demonstrated that miR-155 is expressed in hematopoietic stem-progenitor cells and mature hematopoietic cells, including monocytes, granulocytes, B-cells, and T-cells (23). Subsequent experiments showed that miR-155 plays an essential role in controlling both myelopoiesis and erythropoiesis from CD34^+^ hematopoietic stem-progenitor cells (24, 25).

The miRNA duplex contains two strands identified with either the suffix -5p (from the 5′ arm of pre-miR; i.e., miR-155-5p) or -3p (from the 3′ arm of pre-miR; i.e., miR-155-3p) (26). One of the strands of the duplex is normally discarded (the passenger strand; annotated *) while the retained strand guides eventual mRNA target selection (the guide strand). Thermodynamic properties of the duplex appear to determine strand selection; the strand with the weakest binding at the 5′-end of the duplex is more likely to become the guide strand. Other key characteristics of miRNA guide strands are a U-bias at the 5′-end and an excess of purines (A/G rich), whereas the passenger strands have a C-bias at the 5′-end and an excess of pyrimidines (U/C rich) (27). However, the preferred guide strand can be changed by a single point mutation in the duplex (28), posttranscriptional modification of the duplex (29), and the type of proteins associated with Ago2 in the RISC complex (e.g., trans-activation response RNA-binding protein versus protein activator of dsRNA-dependent protein kinase) (30). Thus, there is increasing evidence demonstrating that both arms of the pre-miRNA hairpin can give rise to guide miRNAs (31, 32) that are biologically functional. In general, miR-155-3p is considered to be the passenger strand (*), and its expression levels are typically 20-fold to 200-fold lower than miR-155-5p. However, despite this difference in expression level, miR-155-3p (*) can be functional, e.g., following TLR7 ligand stimulation of plasmocytoid dendritic cells (pDCs), miR-155-3p is rapidly upregulated while miR-155-5p is induced at a later stage (33, 34). miR-155-3p acts at an early stage by targeting interleukin-1 receptor-associated kinase 3 (IRAK3/IRAKM) mRNA which is a negative regulator of toll-like receptor signaling, facilitating TLR7-induced IFNα/β production, and the later induction of miR-155-5p (miR-155) terminates this production by targeting TGF-β activated kinase 1/MAP3K7 binding protein 2 (TAB 2) mRNA, a key signaling molecule of TLRs (33, 34). Thus, both strands of the miR-155 duplex are required for an efficiently co-ordinated pDCs response. Most studies have investigated the biology of the miR-155-5p strand and further studies are recommended to investigate the evidence for a role of miR-155*(3p) in the regulation of the immune system and disease.

## The Regulation of miR-155 Expression

miR-155 expression is rapidly increased in response to infection or injury. Inducing factors include pathogen-associated molecular patterns and damage-associated molecular patterns (PAMPs/DAMPs (35)), alarmins (e.g., IL-1α) (36), and inflammatory stimuli, e.g., TNF, IL-1β, interferons (35), and hypoxia (37). In contrast, the expression of miR-155 is decreased by anti-inflammatory cytokines, resolvins, glucocorticoids, and posttranscriptional negative regulators, e.g., tristetraprolin; and this decreased expression of miR-155 is an important part of the negative-feedback mechanism terminating immune responses. For example, IL-10 decreases miR-155 expression by inhibiting the transcription factor Ets2. Thus, LPS-induced miR-155 expression is attenuated in Ets2-deficient mice (38). Regulatory cytokines, e.g., TGFβ can induce or inhibit miR-155 expression depending on the cell type and tissue environment (39–41). Resolvins are lipid mediators produced, e.g., by tissue macrophages, upon activation of Tyrosine-protein kinase Mer (MerTK) by apoptotic inflammatory cells (42) and have broad anti-inflammatory effects. Resolvin D1 reduces inflammation in experimental corneal immunopathology by inhibiting miR-155 expression (43). Natural and synthetic glucocorticoids are highly effective at terminating acute inflammation, mediated in part by inhibition of miR-155 expression in a glucocorticoids receptor- and NF-κB-dependent manner (44, 45). Inflammation can be controlled by the short half-life of mRNA of pro-inflammatory mediators (e.g., TNF, GM-CSF, IL-8, and CCL2). The rapid elimination of these mRNAs is mediated by miRNAs; or by specific proteins, e.g., tristetraprolin (TTP/ZFP36) that recognizes adenine–uridine rich elements (AREs) in mRNA and orchestrate its degradation. TTP inhibits miR-155, albeit by an unusual mechanism. Cells that overexpress TTP show high levels of miR-1 that putatively prevents the processing of miR-155 precursor to the mature form (46). In addition, the functions of mature miR-155 are counterbalanced by other miRs and the best example is miR-146. This, induced by the same stimuli as miR-155, targets TRAF6 and IRAK1 of the TLR/IL-1R signaling pathway and the timing of this provides feedback inhibition of the inflammatory response driven by miR-155 (47). This is evident in miR-146-deficient mice that develop inflammatory syndrome, autoimmunity, and cancer. miR-146-deficient cells show an increased expression of miR-155 and their pro-inflammatory phenotype could be normalized by deletion of miR-155 (48), thus miR-155 and miR-146 can cross-regulate inflammatory responses.

The miR-155 gene contains binding sites for multiple transcription factors including sites localized downstream and upstream of exon 1, e.g., 2 NF-κB binding sites (−1,697 and −1,150 bp from the transcription initiation site, respectively), SMAD4 (−600 bp), interferon-sensitive response element ISRE (−311 bp), interferon regulatory factors IRF (−200 bp), and AP-1 (−40 bp). There are also two Ets binding sites within the transcription start site, two Foxp3 binding sites in intron 2, and three hypoxia-inducible factor-1 alpha binding sites in the promoter region (37, 49). This reflects the role of miR-155 in co-ordination of the cell response to changes in the tissue environment (49).

## The Functions of miR-155 in Innate Immune Cells and Its Deregulation in RA

### miR-155 Regulates Polarization of Macrophages

Macrophages can be polarized into different phenotypes in response to changes in tissue environments (50). For example, during bacterial infection, TLRL- or T-helper 1 (Th1)/ILC1-derived IFNγ polarizes the pro-inflammatory macrophage phenotype that drives the protective immune response. However, if uncontrolled, this leads to chronic inflammation (50). Homeostasis can be restored by IL-10 and TGFβ derived from regulatory T-cells and by glucocorticoids, that induce macrophage phenotypes that mediate resolution of the immune response and facilitate tissue repair. IL-4/13 derived from Th2/ILC2 induces alternative macrophage phenotypes that are key in eliminating the parasites (51). However, they are also involved in mediating tissue repair by producing collagen components (proline), TGFβ, and CCL18. These changes in macrophage phenotypes are co-ordinated by miRNA networks, including miR-155 (52).

miR-155 is induced in monocyte/macrophages upon TLR/IFNγ stimulation and drives their inflammatory response by epigenetic regulation of mRNA targets that are inhibitors of innate cell activation, e.g.,

(i) phosphatidylinositol-3,4,5-trisphosphate 5-phosphatase 1 (SHIP-1); an inhibitor of TLR/PI3/Akt kinase pathways.
(ii) SOCS-1; a type 1 cytokine receptor/STAT pathway inhibitor.
(iii) Bcl6, an inhibitor of NF-κB (53–56).

Bone marrow-derived murine macrophages deficient for miR-155 express decreased levels of pro-inflammatory cytokines following LPS stimulation (56, 57), as do miR-155-deficient murine RAW264.7 macrophages (58) and human macrophages gene-silenced for miR-155 (57); each associated with commensurate upregulation of SHIP-1.

**Figure 1:**
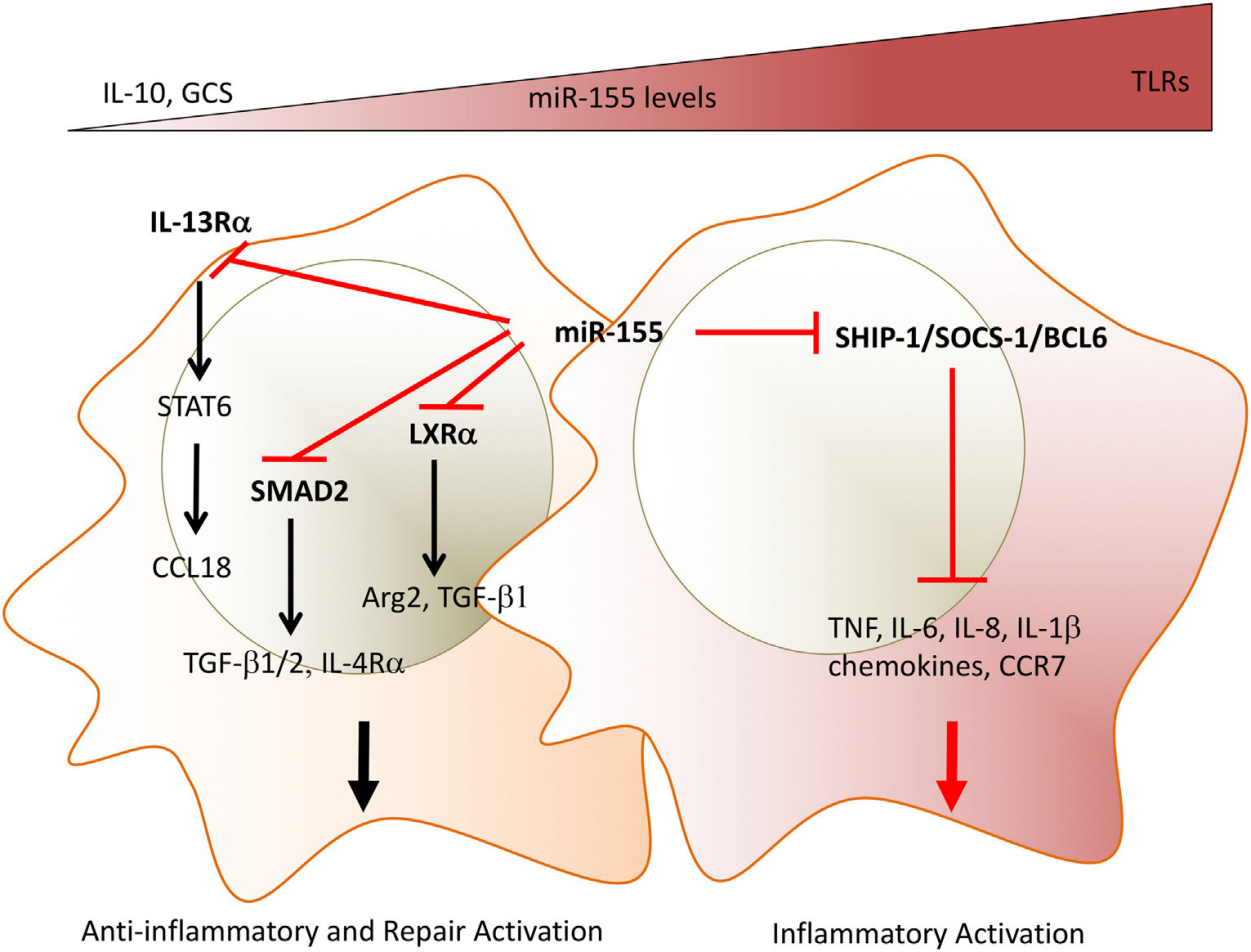
miR-155 acts as a molecular rheostat to determine the duration of inflammation and the initiation of repair programs in macrophages. Depending on the expression levels of miR-155, macrophages can show opposite phenotypes. TLRLs induce high expression levels of miR-155. MiR-155 targets inhibitors of TLR/cytokine receptor pathways and drive a pro-inflammatory phenotype as shown by the production of TNF, IL-1β, IL-6, and chemokines. In addition, high miR-155 levels prevent a phenotypic switch to anti-inflammatory/repair macrophages by targeting (i) IL-13Rα, thus decreasing STAT6 activation and expression of STAT6-dependent mediators, e.g., collagen production, inducing CCL18; (ii) TGFβ signaling molecule SMAD2, and the transcription factor LXRα, thus maintaining low levels of resolution/repair mediators, e.g., TGFβ, Arg2. Glucocorticoid and IL-10 override miR-155-induced phenotype by inhibiting its expression. The chronicity of synovial inflammation may be mediated by persistently high expression levels of miR-155 observed in synovial macrophages. Direct miR-155 targets are marked in bold font. TLR, toll-like receptors; IL-13Rα, interleukin-13 receptor alpha; TGFβ, transforming growth factor beta; SMAD2, small mothers against decapentaplegic homolog 2, LXRa, liver X receptor alpha; Arg2, arginase 2; GCS, glucocorticoids.

In contrast to pro-inflammatory activation of macrophages, miR-155 inhibits the polarization of anti-inflammatory and repair macrophage phenotypes. miR-155 targets translation of multiple molecules in the IL-13/IL-4 pathway, including IL-13Rα, CEBP/β (59), and Mafb (60) and thus prevents development of the STAT6-driven anti-inflammatory and helminth-expulsive macrophage phenotype (61). Furthermore, miR-155 regulates the TGF-β signaling pathway, which is crucial for wound healing and homeostatic remodeling (62, 63). Louafi and colleagues found that miR-155 represses the TGFβ signaling molecule Smad2 in macrophages, preventing TGFβ-induced expression of IL-4Rα, amplification of TGFβ1/2 production, and the development of repair macrophages (63). We recently extended these findings by showing that miR-155 negatively regulates remodeling pathways in alveolar macrophages by targeting the transcription factor LXRα. miR-155-deficient mice had exacerbated bleomycin-induced lung fibrosis compared to WT mice given bleomycin, and their alveolar macrophages showed an increased expression of *Arginase 2*, which is key for collagen production (36). Thus, miR-155 is a master-switch that determines the relative dynamics of TLR-induced inflammatory and IL-13/IL-4 or TGFβ-induced anti-inflammatory/repair phenotypes in macrophages (Figure 1). Inhibition of miR-155, e.g., by glucocorticoids or by regulatory T-cell-derived mediators (e.g., IL-10) allows the emergence of anti-inflammatory and tissue-repair macrophage phenotypes.

### miR-155 Underlies the Activation of Monocytes and Macrophages in RA

Animal studies show that miR-155 drives experimental models of arthritis and its comorbidity, atherosclerosis (56, 57, 64). In RA patients, blood monocytes have significantly higher copy-numbers of miR-155 than healthy controls, and the copy-number correlates strongly with disease activity score (DAS of 28 joints; DAS28) and with erythrocyte sedimentation rate; a non-specific biomarker of inflammation (65, 66). The expression levels of miR-155 in patients in drug-induced remission and with low DAS were similar to levels in healthy donors, whereas levels in patients with medium and high DAS were progressively increased. The expression of miR-155 in monocytes from patients with coronary heart disease is higher than normal and correlated with expression of TNF and IL-6 (67), suggesting that miR-155 expression in monocytes is closely associated with the degree of inflammatory activation (65).

Monocytes are recruited from the blood to the synovial fluid and to the synovial membrane where monocytes differentiate locally into pro-inflammatory macrophages (51, 68). This is an important step in RA progression and is mediated by locally produced chemokines (69). Both CD14^+^ monocytes from synovial fluid and CD68^+^ synovial tissue macrophages from RA patients with active disease have an activated phenotype (70–73) and produce pro-inflammatory cytokines, including TNF, IL-6, and GM-CSF that are targets for current successful RA therapies (74–76). Synovial fluid-derived monocytes can also differentiate toward osteoclasts that mediate bone erosion (77, 78) and promote production of the osteoclastogenic and stromal cell-activating cytokine IL-17 by CD4^+^ T-cells which further contributes to bone and cartilage activation (70–72). We found that miR-155 is constitutively upregulated in both RA synovial tissue macrophages and synovial fluid monocytes compared to RA blood monocytes and non-inflammatory osteoarthritis controls (57). We demonstrated that the increased expression of miR-155 in synovial fluid CD14^+^ cells and synovial tissue macrophages was commensurate with reduced expression of SHIP-1, which thereby enabled their inflammatory phenotype, e.g., TNF production (57). In monocytes, miR-155 supports the expression of the integrin CD11a (LFA-1) that interacts with intercellular adhesion molecule-1 on endothelium and facilitates migration of monocytes into inflamed tissue (79). Moreover, monocyte miR-155 drives the production of inflammatory chemokines, including CCL3, CCL4, CCL5, and CCL8, and downregulates the expression of CCR2 (65). These findings suggest that high levels of miR-155 in pro-inflammatory macrophages in synovial fluid and synovial tissue enables chemokine production and integrin expression that recruits precursors from the circulation, and then mediates the retention of these cells at sites of inflammation by downregulating chemokine receptor expression, and finally mediates local production of pro-inflammatory cytokines. Similarly, miR-155 supports the development of advanced atherosclerotic lesions. In experimental models, miR-155, induced by inflammation and oxidized lipids, targets Bcl6 in lesional macrophages and increases CCL2 production and recruitment of monocytes to vascular walls. Thus, miR-155 deficiency in macrophages ameliorated plaque formation (56).

Blood and synovial fluid monocytes from RA patients are resistant to spontaneous apoptosis and to apoptosis mediated by an agonistic anti-Fas antibody, as compared to blood monocytes of healthy donors (80–84). This resistance may contribute to the persistence of inflammatory monocytes and/or macrophages and their perpetuation of joint inflammation in RA. This resistance is due to the progressive inhibition of CASP10 and APAF1 by the increasing levels of miR-155 in RA cells. This was confirmed by miR-155 overexpression in healthy CD14^+^ cells that conferred resistance to spontaneous apoptosis (84). *In vitro*, mimicking the high expression levels of miR-155 in myeloid cells of patients with RA and cardiovascular disease by enforced expression of miR-155 in healthy human monocytes/macrophages demonstrated that miR-155 triggers the spontaneous production of several pro-inflammatory cytokines, including TNF, IL-6, IL-1β, and IL-8, and decreases anti-inflammatory IL-10 production (57, 65, 84). Thus, miR-155 overexpression promotes a pro-inflammatory phenotype in monocytes/macrophages, whereas *in vitro* treatment of RA patients’ synovial fluid CD14^+^ cells with miR-155 antagonist de-repressed SHIP-1, inhibited the production of TNF (57) and restored cellular homeostasis.

Synovitis is driven by cross talk between synovial macrophages with fibroblasts (10) and emerging evidence suggests that miRNAs packaged into macrophage-derived exosomes regulate the function of adjacent cells, including fibroblasts. This has yet to be formally established is human synovial macrophages and fibroblasts; however, an elegant paper by Wang et al. showed that, upon cardiac injury, macrophages secrete miR-155-enriched exosomes that are absorbed by cardiac fibroblasts. miR-155 is released into the fibroblasts cytoplasm, inhibits SOCS-1, and enhances production of pro-inflammatory mediators, including IL-6. Administration of macrophage-derived exosomes containing miR-155 into miR-155-deficient mice exacerbates myocardial infarction (85). Thus, it would be interesting to reveal the contribution of macrophage-derived miR-155 to the pathogenic phenotype of RA synovial fibroblasts.

**Figure 2:**
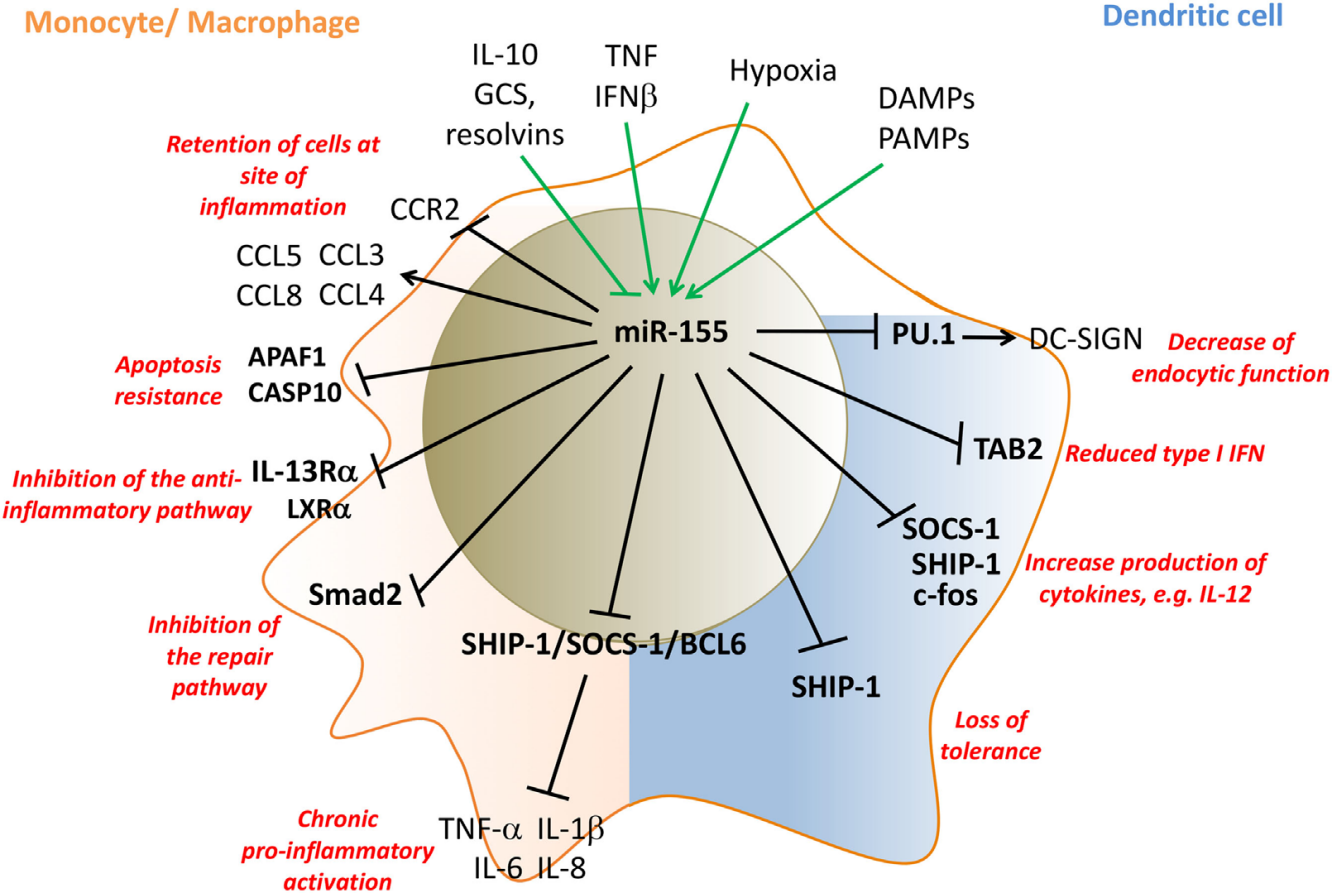
miR-155 regulates pro-inflammatory activation of monocytes/macrophages (pink) and dendritic cells (DCs) (blue). Inflammatory mediators that induce miR-155 expression in innate cells, include LPS, TNF, IFNβ, PAMPs, and DAMPs, while IL-10, GCS, and resolvins inhibit miR-155 expression. miR-155 promotes monocyte recruitment into inflamed tissue by the induction of appropriate chemokines, and then retains them by then downregulating CCR2 and inhibits their apoptosis by targeting APAF1 and CASP10. miR-155 drives inflammatory cytokine production by targeting SHIP-1, SOCS-1, and Bcl6. miR-155 represses the development of anti-inflammatory and repair macrophage phenotypes by targeting IL-13Rα, LXRα, and the TGFβ-signaling molecule SMAD2. In myeloid DCs, miR-155 targets PU.1 and decreases the endocytic function of DCs by downregulating DC-SIGN while supporting maturation of DCs and their T-cell stimulatory activity by targeting SOCS-1/SHIP-1 and c-fos. miR-155 limits plasmocytoid DC activation by decreasing type I IFN production by targeting TAB 2. Direct targets are marked in bold font. LPS, lipopolysaccharides; TNF, tumor necrosis factor; IFN, interferon; DAMPs, danger-associated molecular pattern; CCR2, C–C chemokine receptor type 2; PU.1: Spi-1 proto-oncogene; hematopoietic transcription factor PU.1; CCL, CC chemokine ligands; SHIP-1, phosphatidylinositol-3,4,5-trisphosphate 5-phosphatase 1; SOCS-1, suppressor of cytokine signaling 1; TAB 2, TGF-beta activated kinase 1/MAP3K7 binding protein 2; APAF1, apoptotic peptidase activating factor 1; CASP10, caspase 10; TGF-β, transforming growth factor beta; IL, interleukin; DC-SIGN, DC-specific C-type lectin GCS, glucocorticoids.

In summary, sustained high levels of miR-155 in RA patients’ synovial monocytes and synovial tissue macrophages drive their pro-inflammatory activation and prevent the switch to anti-inflammatory/repair phenotypes, thus preclude resolution of inflammation and initiation of repair (Figure 2).

### miR-155 Regulates Dendritic Cells’ (DCs) Maturation

Dendritic cells are key innate regulators of the adaptive immune response by activating antigen-specific T-cells. They have an important homeostatic function by either mounting an immune response against pathogens or by inducing tolerance to self-antigens. To induce tolerance, DCs activate regulatory T-cells and/or induce anergy of antigen-specific effector T-cells; thereby terminating an immune response and preventing autoimmunity (86, 87). Mouse and human DCs consist of developmentally and functionally distinct DC subsets regulated by a network of cytokines and transcriptional factors (88). Upon stimulation with PAMPs/DAMPs DCs undergo a maturation process which is characterized by functional changes including decreased pathogen binding/endocytic activity, increased cytokine production and enhanced antigen presentation (89, 90). miRNA networks act at checkpoints during the development, differentiation and activation of DC subsets (88). miR-155 is dispensable for DC differentiation but is an important regulator of DC activation; mice deficient in miR-155 have normal numbers of CD8a^+^cDCs, CD8a^−^cDCs, and pDCs (91); however, miR-155 is readily induced in mouse and human DCs following TLR stimulation (35, 92, 93). TLR stimulation activates two cellular pathways resulting in DC maturation; MyD88-IRAK-TRAF6 and TRIF-IRF3-IFNα, and both are required for optimal NF-κB activation, expression of co-stimulatory molecules, and the production of cytokines, such as IL-1β or IL-12 (94, 95). miR-155 regulates the switch from the endocytic function to co-stimulatory/activatory function during DC maturation. miR-155 reduces the capacity of DCs to bind pathogens by downregulation of DC-SIGN *via* inhibition of transcription factor PU.1 (93), while enhancing cytokine production (91, 96, 97). Studies show that miR-155^−/−^ DCs exhibit lower levels of MHC class II and co-stimulatory molecules (CD80, CD40), and a decrease in *IL1*β, *TNF, IL12*, and *IL6* expression upon TLR-induced maturation compared to wild-type DCs, and they demonstrate a significantly lower ability to induce effector T-cells (91, 98). This may be mediated by miR-155 inhibition of c-fos, continued expression of which was shown to be detrimental for DC maturation (91). Similarly, inhibition of miR-155 in human monocyte-derived DCs caused a reduction in IL-12p70 production (97) whereas overexpression of miR-155 in mature DCs enhanced *IL12p70* expression, leading to higher levels of IFN-γ being released by cocultured natural killer cells (97). It was proposed that miR-155 mediated these activatory functions of DCs through epigenetic regulation of SHIP-1 and SOCS-1 in a similar manner as in macrophages (96, 97).

Interestingly, miR-155 may terminate some DC functions by targeting TAB 2, a key adaptor molecule in the TLR/IL-1 pathway. These include termination of type I IFN or IL-1β production induced by TLR7- or LPS-mediated human monocyte-derived DC activation, respectively (55, 92). This discrepancy requires further investigation, but the nature of the stimulatory factor and the abundance of specific target at precise time–points during DC activation may determine whether miR-155 supports or terminates DC activation.

### The Role of miR-155 in RA DC Activation

Rheumatoid arthritis peripheral blood, synovial fluid and synovial tissue DCs, in particular CD1c^+^ and monocyte-derived populations, show a constitutively activated phenotype by their increased expression of co-stimulatory molecules, cytokines (e.g., *IL6*) and their ability to activate autologous T-cells (99–102). It was shown recently that high levels of miRNA-34a that lead to repression of Axl, an inhibitor of DC activation, contribute to the activated phenotype of circulating and synovial CD1c^+^ in RA (100). The involvement of miR-155 to this phenotype is unknown. However, a role for DC miR-155 in driving experimental autoimmunity was shown by Lind et al. (96) in a model of diabetes in which self-antigen-pulsed, TLR-matured DCs lacking miR-155 have an impaired ability to break immune tolerance, while the transfer of self-antigen-pulsed DCs overexpressing miR-155 was sufficient to break tolerance even in the absence of TLR stimuli (96).

## miR-155 Functions in Adaptive Immune Cells and is Deregulated in RA

Several lines of evidence support the role of miR-155 in T- and B-cell differentiation. miR-155 controls both effector and regulatory T-cell function (103) and regulates B-cell proliferation, germinal center formation, and antibody production (104). If aberrantly expressed, miR-155 contributes to initiation and progression of malignancies of B-cells and to autoimmunity.

### miR-155 Controls B-Cell Proliferation

miR-155 is highly expressed in human B-cell lymphomas, especially in large B-cell lymphomas, Hodgkin lymphomas, and certain types of Burkitt lymphomas (104). Transgenic mice expressing miR-155 under the control of Eμ enhancer and VH promoter (Eμ-miR-155) develop aberrant accumulation of *pre-B-cells* leading to the development of acute lymphoma. These cells have commensurately decreased expression of miR-155 targets, e.g., SHIP-1 and CCAAT enhancer-binding protein beta (C/EBPbeta), which in B-cells have anti-inflammatory and anti-proliferative functions (105, 106). miR-155 may promote survival and proliferation of *naïve B cells* in Eμ-miR-155 mice by targeting co-repressor partners Hdac4 (directly) and Bcl6 (indirectly), and their reduction leads to de-repression of Bcl6 targets, e.g., inhibitor of differentiation-2, IL-6, cMyc, Cyclin D1, and Mip1α/ccl3; all of which promote cell survival and proliferation. Importantly, meta-analysis of microarray data from diffuse large B-cell lymphoma patients found that miR-155 expression correlated negatively with Bcl6 and Hdac4 expression, strongly supporting the role of the miR-155/Bcl6/Hdac4 pathway in the pathogenesis of human leukemias (107). miR-155 also regulates proliferation of *mature B-cells* in germinal centers by interfering with TGF-β signaling. TGF-β1 secreted in secondary lymphoid organs limits mature B-cell proliferation to maintain immune-system homeostasis. miR-155 represses the TGF-β signaling molecule SMAD5, resulting in defective expression of the cell-cycle inhibitor p15/p21 (108) and allowing B-cells unhindered progress through the cell-cycle.

Systemic delivery of antisense miR-155, encapsulated in polymer nanoparticles, inhibited miR-155 and slowed the growth of pre-B-cell tumors *in vivo*, confirming that miR-155 is an important regulator of B-cell proliferation, and this may be a promising therapeutic option for lymphoma or leukemia (109).

### miR-155 Controls Antibody Production by B-Cells

A critical stage of effector B-cell development is represented by germinal center (GC) formation. These structures develop when mature naïve B-cells encounter cognate antigen in the secondary lymphoid organs during which the B-cells undergo somatic hyper-mutation of their immunoglobulin variable region genes and class-switch recombination. Emerging B-cells that express high-affinity antibody further differentiate into plasma cells and memory B-cells (110). B-cells also contribute to the immune response by acting as antigen-presenting cells and fuel inflammation by releasing a wide range of cytokine and chemokines (10). Thai and co-workers demonstrated that miR-155 plays an essential role in the regulation of GC formation. They found that miR-155-deficient mice had greatly reduced GC formation that was due to reduced production of TNF and LTα; obligate cytokines for GC neogenesis, by B-cells in response to antigen-ligation of the B-cell receptor (103). In support of a pivotal role for miR-155 in B-cell activation, chronic lymphocytic leukemia (CCL) B-cells that expressed high miR-155, demonstrated miR-155-dependent increased activation (calcium flux) induced by BCR ligation (111). The *in vivo* antibody response of miR-155-deficient mice showed significantly reduced concentration of IgM and isotype-switched antigen-specific antibody class production (98, 112). Mechanistic studies by Vigorito et al. (112) showed that miR-155 can directly target PU.1 mRNA *via* a predicted miR-155-binding site in the 3′ UTR, and that miR-155-deficient B-cells had increased PU.1 expression. Enforced expression of PU.1 in wild-type primary B-cells led to a reduction in the proportion of IgG1-expressing cells upon stimulation (112, 113). Mechanistically, miR-155 reduces PU.1 expression that leads to the downregulation of Pax5, a PU.1-dependent inhibitor of B-cell activation. This enables plasma cell differentiation along with the expression of other genes involved in B/T cellular interactions (e.g., Sema4a, Sema4b, Sema7a, and CD300If) facilitating antibody production (114).

miR-155 can also regulate the antigen-presenting function of B-cells. miR-155 targets the 3′UTR of CD1d in B-cells (115). Therefore, upregulation of miR-155 in B-cells, e.g., in chronic inflammation, can repress CD1d leading to impaired lipid-antigen presentation by B-cells to invariant natural killer cells. This causes a breach of tolerance as shown by CD1d deficiency exacerbating lupus nephritis in the pristine-induced model (115).

Of additional relevance to autoimmune disease, miR-155 modulates the B-cell response to immune complexes. The low-affinity IgG inhibitory receptor FcγRIIB (Fcγ receptor IIB) is key in terminating proliferative signals delivered by autoantigen-containing immune complexes by activating SHIP-1. Thai et al. showed that miR-155 can downregulate SHIP-1 and permit immune-complex-induced pathology in the Fas^lpr^ model of autoimmune lupus (113), and this may also be relevant to the signaling of the immune complexes in RA.

### The Role of miR-155 in RA B-Cells

The onset of RA is preceded by preclinical immunological changes, e.g., the production of anti-citrullinated peptides antibodies (anti-CCP/ACPA) and rheumatoid factor, which reflect the loss of immunological tolerance to self-antigens (116), and this places B-cells at the center of RA pathogenesis. This is confirmed by the efficacy of B-cell depletion therapy in RA patients (117). The proportion of B-cell subsets is altered in the blood of RA patients (118, 119) with higher percentages and absolute numbers of naïve B-cells at the onset of disease, and increased proportions of double-negative (IgD^−^/CD27^−^) memory B-cells and plasmablasts in patients with established RA (120).

**Figure 3:**
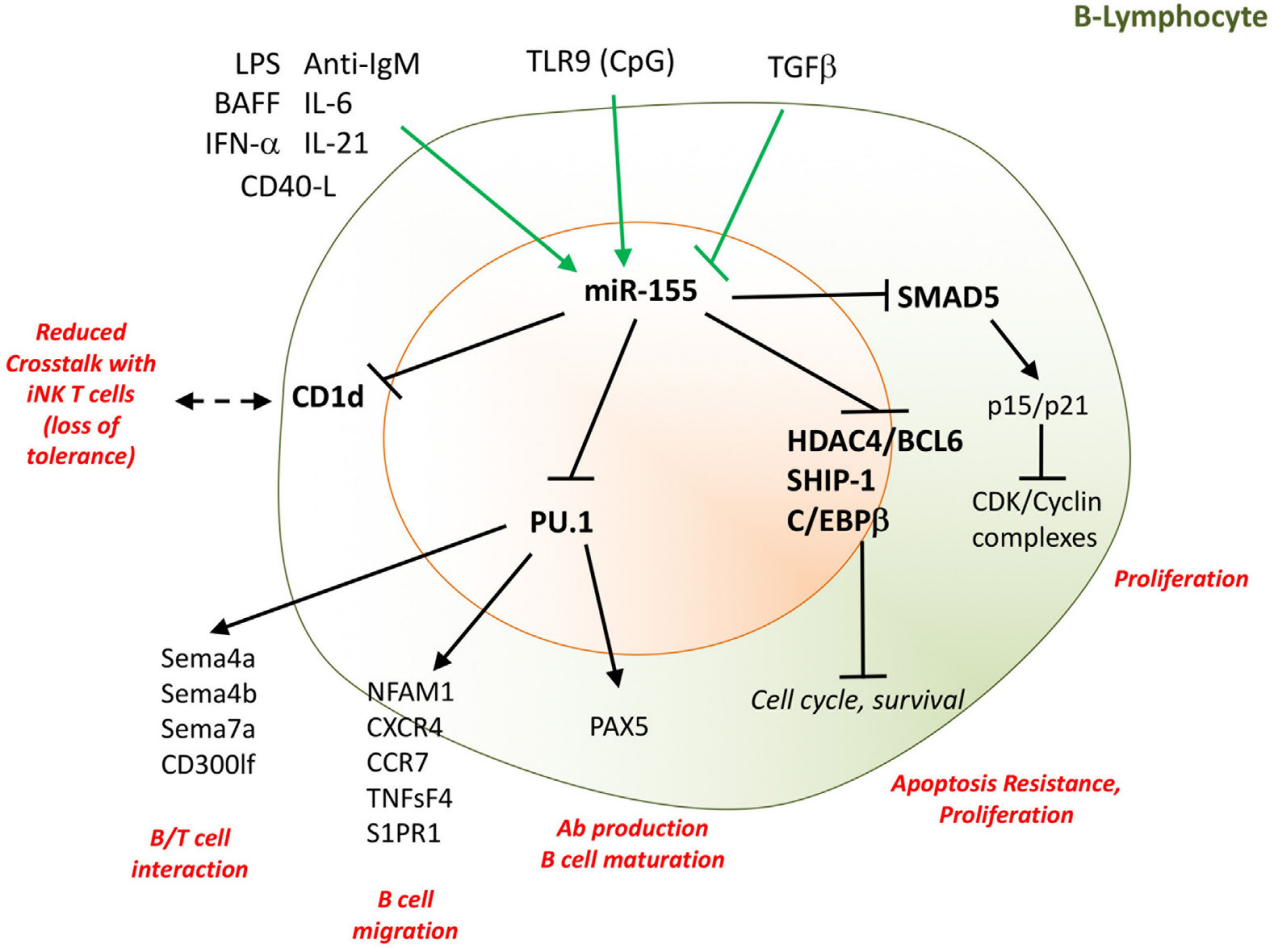
miR-155 supports B-cell proliferation and antibody production. Several cytokines and TLR-ligands can induce miR-155 expression in B-cells. These include LPS, IL-6, IL-21, CD40L, IFN-α, and BAFF, while TGFβ inhibits miR-155 expression. By targeting SHIP-1, C/EBPβ, the Hdac4/Bcl6 complex, and the TGFβ signaling molecule SMAD5, miR-155 drives proliferation and resistance to apoptosis of B-cells. miR-155, by epigenetic regulation of PU.1, is indispensable for B-cell maturation, migration, interaction with T-cells, and antibody production. Moreover, miR-155 regulates the surface expression of CD1d on B-cells, influencing the cellular cross talk with invariant NK T-cells. Direct targets are marked in bold font. LPS, lipopolysaccharides*;* IL, interleukin; TLR, toll-like receptor; Bcl, B-cell lymphoma; SMAD5, small mother against decapentaplegic 5; CD, cluster designation; PU.1, Spi-1 Proto-Oncogene; Sema, semaforin; NK, natural killer; BAFF, B-cell activating factor; CD40L, CD40 ligand. TGF-β, transforming growth factor beta; SHIP-1, phosphatidylinositol-3,4,5-trisphosphate 5-phosphatase 1; Bcl6, B-cell lymphoma; HDAC4; histone deacetylase 4; C/EBPβ: CCAAT/enhancer-binding protein beta; NFAM1, NFAT activating protein with ITAM motif 1; CXCR4; C–X–C chemokine receptor type 4; CCR7, C–C chemokine receptor type 7; S1Pr1, sphingosine-1-phosphate receptor 1; TNFsF4, tumor necrosis factor (ligand) superfamily, member 4; CD300lf, CMRF35-like molecule 1.

As previously stated, miR-155 was first described as an important epigenetic regulator of antibody synthesis (98, 112). In experimental models of arthritis, the production of anti-collagen antibodies was significantly decreased in miR-155-deficient mice compared to wild type (57, 64), and these mice did not develop arthritis. In RA patients, we found that the expression of miR-155 in B-cells is increased and is maximal during the early phase of disease. This increase is predominant in double-negative (IgD^−^/CD27^−^) memory B-cells (121), and occurs in ACPA^+^ but not ACPA^−^ RA patients suggesting a role in autoantibody production. The IgD^−^/CD27^−^ B-cell population is increased in other autoimmune diseases, including SLE (122, 123) and is important for IgG^+^ plasmablast generation (124). In a study of paired peripheral blood–synovial fluid samples from RA patients, miR-155 expression was higher in B-cells from the synovium compared with B-cells from blood. Mediators, including IL-6, BAFF, IgM, CD40L, and IL-21 were increased in synovial fluid, and these were able to upregulate miR-155 expression in B cells *in vitro*. In addition, *in situ* hybridization revealed that most of the resident synovial tissue CD20^+^ cells in germinal-center-like follicles were miR-155 positive. Commensurate with the increased miR-155 expression, synovial fluid-derived B-cells of RA patients showed reduced expression of the miR-155 target PU.1 in ACPA^+^ compared to ACPA^−^ patients, and a reduced proportion of PU.1^+^ cells among the resident CD20^+^ cells in synovial follicles (121). Inhibition of endogenous miR-155 in RA B-cells led to the restoration of PU.1 expression and inhibition of antibody production (121) suggesting a key role of miR-155 in autoantibody production by B-cells in RA. Commensurate with this, the expression of miR-155 in circulating B-cells of ACPA^+^ RA is significantly higher than that of similarly inflamed patients with psoriatic arthritis (PsA), in which B-cells are minimally involved in pathogenesis (121). In addition, the expression of miR-155 in circulating B-cells is particularly high in RA patients with follicular synovitis; which is a prognostic factor for poor treatment response (125). Thus, miR-155 expression in RA circulating B-cells can serve as a biomarker of B cell activation and lymphoid/follicular synovitis among the three RA synovial pathotypes identified (myeloid, lymphoid/follicular, and fibroid) (126). A summary of miR-155 function in B-cells is presented on Figure 3.

### miR-155 Is a Regulator of T-Cell Differentiation

In response to appropriate stimuli, CD4^+^ T-cells can differentiate into different phenotypes that include the Th1, Th2, Th17, follicular helper T (Tfh) cells, and T-regulatory (Treg) cell lineages with distinct functions (127). This differentiation is influenced by cytokines and by the expression and activation of phenotype-specific transcription factors (128). miRNAs have been implicated in CD4^+^ T-cell differentiation (129, 130) and miR-155 is central to this process (98, 131).

#### T-Helper 1

Rodriguez and colleagues examined the T-cell response of miR-155-deficient mice immunized with T-dependent antigen (tetanus toxin fragment C) and showed that splenocytes produced significantly reduced concentrations of IL-2 and IFN-γ compared to wild-type mice (98). An intrinsic requirement for miR-155 to Th1 development was confirmed *in vitro* by comparing the cytokine production by WT and miR-155^−/−^ CD4^+^ cells in culture following anti-CD3 and anti-CD28 stimulation, which demonstrated reduced IFNγ production by miR-155^−/−^ cells (98). In keeping with these observations, Banerjee and co-workers showed that overexpression of miR-155 in activated CD4^+^ T-cells promoted Th1 differentiation by targeting IFN-γRα thereby decreasing the sensitivity of Th1 cells to the anti-proliferative effects of IFN-γ (131).

#### Th2

miR-155 was initially described as a negative regulator of Th2 commitment through the repression of its target c-Maf, which is an IL-4 promoter transactivator (98). CD4^+^ T-cells from miR-155-deficient mice exhibit preferential Th2 differentiation upon activation (132–134). miR-155-deficient mice have an age-dependent increased number of Th2 cells and develop spontaneous airway remodeling due to the action of Th2 cytokines (98). In keeping with this, we recently reported that miR-155-deficient mice are susceptible to exacerbated bleomycin-induced lung fibrosis (36). In contrast, Malmhall et al., using an antigen-induced asthma model, observed that miR-155-deficient mice had decreased activation of Th2 cells and decreased production of IL-4, IL-5, and IL-13, decrease lung eosinophilia and fewer physiological changes compared with wild-type mice (135). The potential inhibition of Th2 development was not associated with c-Maf changes but with depression of PU.1 that negatively regulates the expression of GATA3, the key transcription factor for Th2 cell function. Similarly, Okoye et al. described a Th2-cell-selective role for miR-155 in the development of an allergic airway model. They demonstrated that miR-155 inhibits the expression of sphingosine-1-phosphate receptor 1, which is required for lymphocyte egress from lymphoid organs, thus mice with miR-155^−/−^ T-cells had reduced airway pathology (136). This suggests that the miR-155 may highlight different roles for Th2 responses in acute allergic inflammation compared with fibrosis. It is possible that resident tissue memory T-cells are more involved in age-related and bleomycin-induced chronic lung remodeling, whereas recently activated recruited T-cells are more involved in acute airway inflammation.

#### Th17

Th17 lymphocytes have higher expression levels of miR-155 compared with other T-cell subset lineages (137). miR-155 is upregulated in Th17 cells by STAT3, IRF-4 and RORγt and supports the development of Th17 by inhibiting its epigenetic target Jarid2, which itself is an epigenetic suppressor of Il22, Il10, Il9, and Atf3 (137), and by suppressing SOCS-1, thereby facilitating IL-6/STAT3 signaling (138). Accordingly, miR-155-deficient mice have substantially reduced numbers of Th17 cells as demonstrated by reduced expression of IL-17A (139), IL-22, IL-6, and IL-23R (64, 139–141).

#### Follicular T-Helper Cells (Tfh)

Follicular T-helper cells cells are key in GC formation and antibody production by providing help to B-cells in secondary lymphoid tissue. Elegant work led by Tang showed that miR-155 is indispensable in the development of Tfh. Using CD4^+^-specific miR-155-deficient mice, the authors showed reduced levels of Tfh (CXCR5^+^PD1^+^ Bcl6^+^) and consequently less-developed GCs and reduced antigen-specific antibody production in a variety of immunization schedules. Silencing of two proteins, Fosl2 (a component of AP1) and to a lesser extent Peli1 (E3 ubiquitin ligase) in miR-155^−/−^ CD4^+^ T-cells, resulted in improved development of Tfh cells following immunization, suggesting that miR-155 mediates Tfh differentiation by downregulating these two targets (142).

#### Regulatory T-Cells

There is abundant evidence that miR-155 can promote the development of Tregs. These cells are important to maintain immunological tolerance by limiting pathogenic Th-cell responses. Tregs are dependent on IL-2/STAT5 signaling and are characterized by the expression of Foxp3. This transcription factor induces miR-155 which then upregulates STAT5 phosphorylation by its epigenetic blockade of SOCS-1-mediated inhibition of STAT5. miR-155-deficient mice have reduced numbers of Tregs in the thymus and in peripheral blood due to attenuated IL-2 signaling by de-repressed SOCS-1 (143, 144). Cytotoxic T-lymphocyte antigen 4 (CTLA-4), which is an effector molecule of Tregs that binds to CD28 and limits the activation of effector T-cells, is a putative mRNA target of miR-155 (143, 145). However, Tregs from miR-155-deficient mice express normal levels of CTLA-4 suggesting that it is not under the epigenetic control of miR-155, at least in Tregs in mice. In contrast, Sonkoly et al. showed that miR-155 targets CTLA-4 mRNA in human effector T-cells. This group observed an increased level of miR-155 and decreased level of CTLA-4 in effector T-cells of patients with atopic dermatitis, suggesting that miR-155 inhibition of CTLA-4 contribute to activation of effector T-cells in the skin (146). Thus, miR-155/CTLA-4 interactions might be cell-type and species-specific.

#### CD8^+^

Deletion of Dicer in cytotoxic CD8^+^ T-cells causes an impaired immune response to pathogens (147). Gracias et al. (148) found miR-155 upregulated in primary effector and effector memory CD8^+^ T-cells as compared to naive and central memory CD8^+^ cells. Antiviral CD8^+^ T-cell responses and viral clearance were impaired in miR-155-deficient mice, while miR-155 overexpression augmented antiviral CD8^+^ T-cell responses *in vivo*. Increased miR-155 inhibited the type I interferon response in CD8^+^ cells while miR-155-deficient CD8^+^ T-cells had enhanced type I interferon signaling and were more susceptible to IFNs antiproliferative effect. Inhibition of type I interferon-associated transcription factors STAT1 or IRF7 resulted in enhanced responses of miR-155-deficient CD8^+^ T-cells to viral infection *in vivo* (148). This suggests that miR-155 maintains proliferation of CD8^+^ in response to viral antigen by downregulation of STAT1/IRF7.

### The Role of miR-155 in T Cells in RA

In experimental arthritis, miR-155-deficiency affects Th17 cell polarization. Production of IL-17 and IL-22 are significantly reduced in miR-155-deficient mice compared to wild type (64). Smigielska-Czepiel and colleagues provided important insight into the differential expression of specific miRNAs in human T-cell subsets, especially naive and memory T-cells in peripheral blood and synovial fluid in RA patients (149). Synovial fluid T-cells are predominantly memory T-cells (150) and are characterized by high expression of miR-155 compared with naive T-cells (149), thus miR-155 in this compartment may support local production of IL-17 and IFNγ by autoreactive T-cells.

Despite the presence of many Tregs in the synovial fluid of RA (151–153), joint inflammation persists in most patients regardless of the treatment regimen. One interpretation is that there is a functional defect in these Tregs, e.g., due to inhibition of Foxp3 by TNF (154) that is in high concentration in synovial fluid; or that the effector T-cells are resistant to the regulatory action of Tregs (155). Blood Tregs from RA patients have significantly lower expression of miR-155 than healthy controls (156). However, correcting this expression by enforcing exogenous miR-155 in RA Tregs *in vitro* enhanced their production of TNF, IL-17, and IFNγ after 24 h upon stimulation with anti-CD3/CD28 (156). This suggests that altering the expression of miR-155 alone is not sufficient to restore the immunosuppressive function of RA Tregs but promotes pathogenic cytokine production instead.

**Figure 4:**
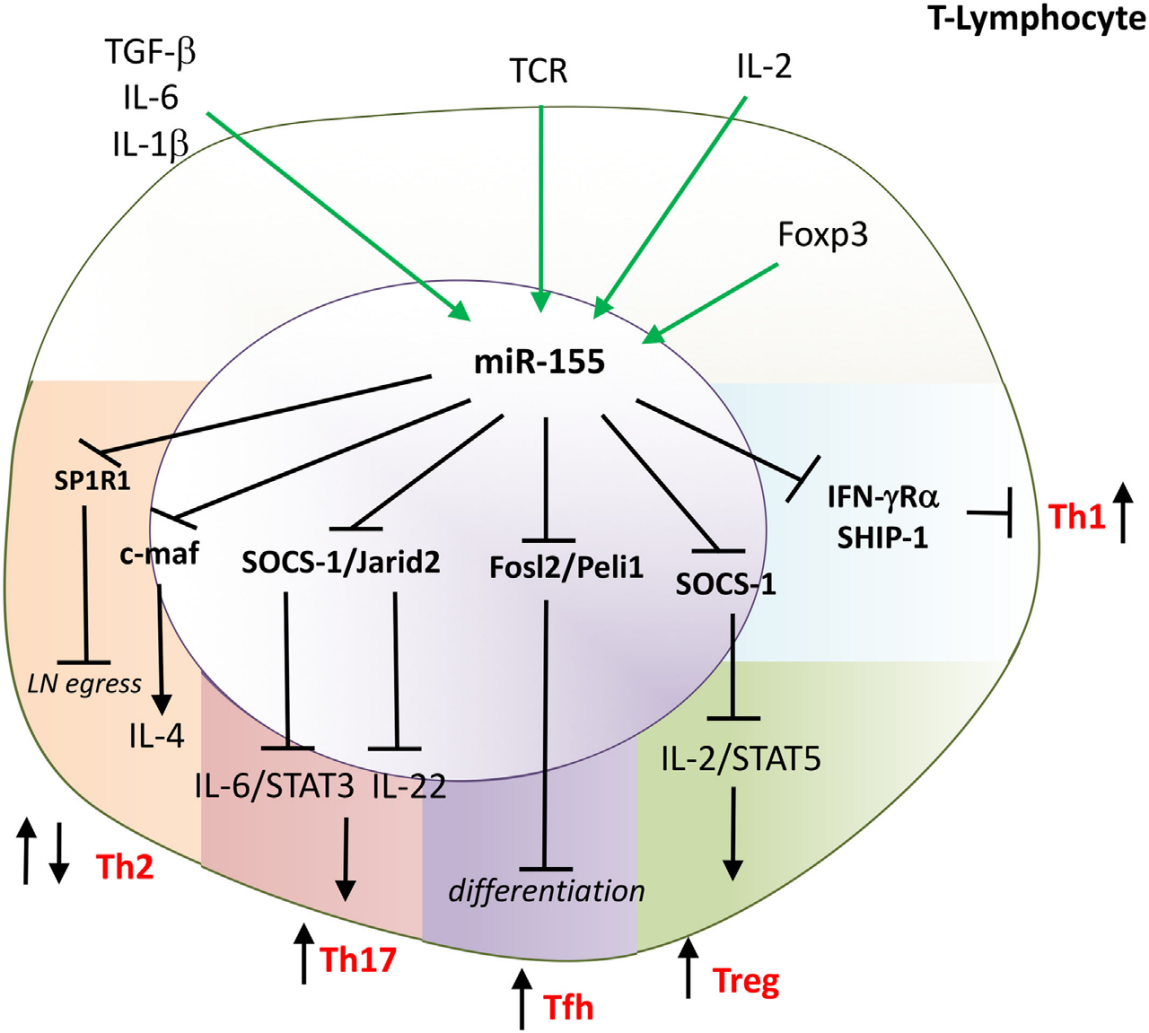
miR-155 drives differentiation of T-helper 1 (Th1), Th17, Tfh and is indispensable for the function of Tregs. miR-155 has multiple effects on T-cell function. miR-155 promotes: Th1 by targeting IFNRα; Th17 by targeting Jarid2 and SOCS-1; and follicular helper T-cells (Tfh) by targeting Fosl2 and Peli. Mir-155 supports the function of regulatory T-cells (Treg) by targeting SOCS-1 that enables IL-2 signaling. miR-155 regulates Th2 development and function; e.g., inhibiting development by targeting c-maf; a key transcription factor for IL-4 and regulating an egress of T cells from the lymph nodes by targeting S1PR1. Direct targets are marked in bold font. TGF, transforming growth factor; IL, interleukin; TCR, T cell receptor; IFN, interferon; SOCS-1, suppressor of cytokine signaling 1; STAT, signal transducer and activator of transcription; SHIP, phosphatidylinositol-3,4,5-trisphosphate 5-phosphatase 1; c-maf, transcription factor Maf; reg, regulatory; JARID2, Jumonji, AT rich interactive domain 2; SP1R1, sphingosine-1-phosphate receptor-1.

In summary, miR-155 is indispensable for activation of Th1, Th17, Tfh, CD8^+^, and Treg while inhibiting Th2 (Figure 4). Sustained high expression of miR-155 in synovial effector T-cells may contribute to chronic production of IFNγ, IL-17 and facilitate differentiation of T-cells toward Tfh cells in RA.

## Is There Therapeutic Potential in Targeting miR-155 in Arthritis?

**Figure 5:**
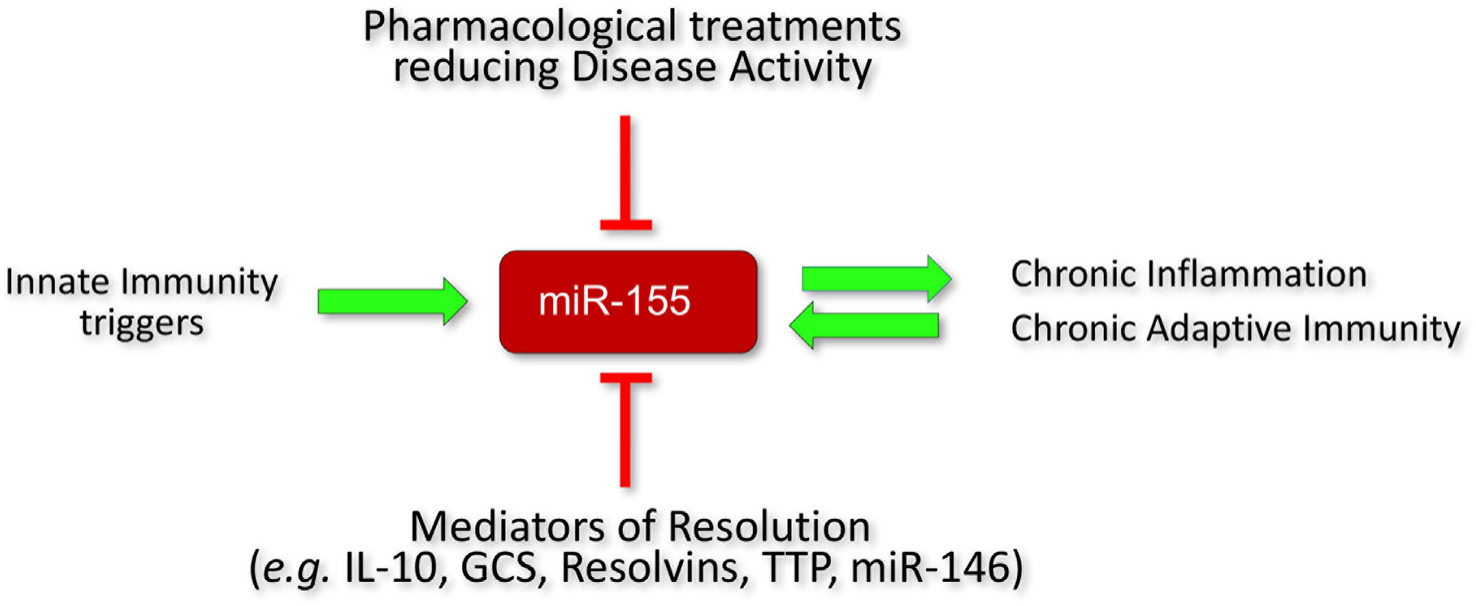
Regulation of miR-155 expression in RA. Initiators of inflammation, e.g., PAMPs and DAMPs increase miR-155 expression. Homeostatic resolution is controlled by anti-inflammatory mediators that act in part by decreasing miR-155 expression (e.g., IL-10, GCS, TTP, resolvins) or counterbalancing its function, e.g., miR-146. In rheumatoid arthritis (RA), there is persistent inflammation and activation of adaptive immunity which maintain high expression levels of miR-155 that lock the immune system in an activatory state. A successful response to treatment (reduced systemic and synovial inflammation) in RA patients is associated with a reduction of miR-155 expression (at least in monocytes). PAMPs, pathogen-associated molecular patterns; DAMPs, damage-associated molecular patterns; GCS, glucocorticoids; TTP, tristetraproline; cDMARD, conventional disease-modifying anti-rheumatic drugs; TNFi, TNF inhibitors.

The literature and our own experimental findings summarized in this review indicate that miR-155 is a key mediator of chronic activation of innate and adaptive immunity in RA. Its contribution to RA pathogenesis might be more profound than in other forms of inflammatory arthritis such as PsA due to the involvement of miR-155 in driving autoantibody production in RA. A cross-sectional analysis of RA patients with different clinical responses to treatments revealed that miR-155 expression becomes reduced in the monocytes of RA patients who responded well to conventional disease-modifying anti-rheumatic drugs (e.g., methotrexate) or to biological DMARDs (65). Consistent with this, there are prospective studies in psoriasis patients showing that the expression levels of miR-155 in PBMCs decreased upon successful treatment with methotrexate as compared to pre-treatment levels (157). These observations suggest that a reduction of high to normal expression of miR-155 reflects a good clinical response to therapies that reduce inflammation (Figure 5). However, the effects of current anti-inflammatory (e.g., *anti TNF, anti-IL-6R*) or anti-adaptive immunity (e.g., *CTLA-4*) therapies on miR-155 expression in B-cells and Th17 cells still needs to be investigated in prospective studies.

Chronic inflammation and continuous activation of adaptive immunity generate pro-inflammatory cytokines and autoantibodies that fuel high levels of miR-155 expression thus locking the immune system into an activatory state. Targeting miRNAs has proven to be a useful therapeutic option in infectious diseases, cancer and recently in tendinopathy (8, 9). The most clinically advanced example of targeting miRNA is neutralization of miR-122 to treat hepatitis C infections (HCV) that is currently in phase IIa clinical trials. miR-122 supports the replication of HCV (158) and treatment with antimiR-122 (Mirvirasen; Roche/Santaris and Regulus Therapeutics) achieved a reduction in viral titers in HCV-infected patients (8). Another miR-targeted therapeutic that reached clinical development is a mimic of the tumor suppressor miR-34. miR-34a targets pro-apoptotic proteins and cell-cycle inhibitors, and is downregulated in many human cancers (159). The miR-34 mimic MRX34 (Mirna Therapeutics), encapsulated in lipid nanoparticles, is currently being tested in several solid and hematological malignancies (8). We showed recently that chronic tendinopathy is driven by deregulation of the IL-33/miR-29 pathway leading to the production of pro-inflammatory mediators and collagen 3 with weak tensile strength, thus limiting effective tendon healing (160). The results from a randomized, blinded trial of local tendon miR-29a replacement therapy in an equine model, that closely mimics human disease, changed the collagen composition toward stronger collagen 1 and improved early tendon healing (9). Human trials will be conducted in the near future.

To date, the potential clinical outcome of miR-155 inhibition in arthritis is based on findings from animal models, and from *ex vivo* studies using RA patient-derived cells. miR-155-deficient mice are resistant to arthritis, and inhibition of miR-155 using specific antimiR in a collagen-induced arthritis model reduced the clinical onset and severity of disease (161). Thus, inhibiting miR-155 may represent an attractive therapeutic option. However, systemic inhibition of miR-155 may compromise its other physiological functions and lead to unwanted consequences. Experimental and clinical evidence suggest that miR-155 is indispensable for normal immunity against pathogens (55, 103). In addition, miR-155 in liver macrophages (Kupffer cells) is key in the regulation of lipid metabolism. Mir-155 regulates the expression levels of a target mRNA LXRα which is a transcription factor that controls lipid metabolism and lipid efflux, thus miR-155-deficient mice have an increased level of circulating cholesterol and develop fatty liver (liver steatosis) while on a high-fat diet (162). Furthermore, miR-155 controls lung interstitial remodeling by regulating the proliferation and collagen production of lung fibroblasts, the repair macrophage phenotype, and the production of type 2 cytokines (36, 98). miR-155-deficient mice develop spontaneous age-related lung fibrosis and exacerbated lung fibrosis associated with administration of bleomycin. The lack of miR-155 epigenetic control in these murine lung fibroblasts resembles the deregulated *in vitro* pro-fibrotic behavior of lung fibroblasts from patients with idiopathic pulmonary fibrosis (36, 98). This suggests that a therapeutic strategy in arthritis of systemic targeting of miR-155, or pan-macrophage-specific delivery of miR-155 inhibitors *via* liposomes might have detrimental effects. There may be an alternative method of delivery of a miR-155-inhibitor. An elegant study led by Cheng et al. (163) showed that delivery of anti-miR-155 could be specifically targeted to hypoxic tissues such as tumors or inflamed tissues with an ambient low pH created by anaerobic metabolism. The authors linked antimiR-155 with a low pH-induced transmembrane structure (pHLIP). At pH less than 7, the C-terminus of pHLIP inserts across lipid bilayers and facilitates delivery of the attached antimiR-155. Inside the cell, the disulfide bond between pHLIP and antimiR-155 is reduced in the cytosol and the intracellular antimiR-155 is free to inhibit miR-155. The inflamed synovial tissue environment is hypoxic (164), thus this novel delivery systems could inhibit miR-155 in arthritis and avoid off-target side effects such as reduced protective immunity, fibrosis, or liver steatosis. Alternatively, enhancing activation of inhibitors of cell activation that are repressed by miR-155, e.g., SHIP-1 by specific activators might be a safer option (165). SHIP-1 activator (AQX-1125) has generated positive clinical data from clinical trials in COPD and allergic asthma, demonstrating a favorable safety profile and anti-inflammatory activity (*trial number: NCT01954628*).

## Future Directions

There are gaps in the identification and characterization of all direct miR-155 targets and the physiological impact of miR-155 inhibition on these targets would need to be revealed before anti-miR-155 therapy can be considered. Moreover, understanding the role of miR-155 that is naturally contained in vesicles transferred between cells in the synovium may provide insight into mechanisms of chronicity. Investigation of miR-155 in DCs and B-cells in the preclinical phase of RA (asymptomatic ACPA positive individuals) would help to dissect its role in the regulation of the breach of immunological tolerance. A biomarker for treatment responses in arthritis would be of considerable clinical value, therefore, prospective studies using cohorts of RA patients at different stages of disease would validate whether miR-155 could fulfill this additional role.

## Author Contributions

MK-S and SA selected the research and wrote the review. EG, CM, BT, GF, and IM selected the research.

## Conflict of Interest Statement

The authors declare that the research was conducted in the absence of any commercial or financial relationships that could be construed as a potential conflict of interest.
